# Symmetry function in gait pattern analysis in patients after unilateral transfemoral amputation using a mechanical or microprocessor prosthetic knee

**DOI:** 10.1186/s12984-021-00810-w

**Published:** 2021-01-19

**Authors:** Mateusz Kowal, Sławomir Winiarski, Ewa Gieysztor, Anna Kołcz, Karolina Walewicz, Wojciech Borowicz, Alicja Rutkowska-Kucharska, Małgorzata Paprocka-Borowicz

**Affiliations:** 1 Department of Physiotherapy, Wroclaw Medical University, Grunwaldzka 2, 50-355 Wroclaw, Poland; 2 Department of Biomechanics, University School of Physical Education in Wroclaw, Wroclaw, Poland; 3 Laboratory of Ergonomics and Biomedical Monitoring, Wroclaw Medical University, Wroclaw, Poland; 4 Faculty of Health Sciences, University of Opole, Opole, Poland; 5 Department of Nervous System Diseases, Wroclaw Medical University, Wroclaw, Poland

**Keywords:** Transfemoral amputation, Symmetry function, Gait analysis, Biomechanics, Rehabilitation

## Abstract

**Background:**

Above-knee amputations (AKAs) contribute to gait asymmetry. The level of asymmetry is affected by the type of knee prosthetic module. There is limited evidence suggesting that more technically advanced solutions (microprocessor modules; MicPK) are superior to less advanced ones (mechanical modules; MechPK). The study aimed to evaluate the variable range of hip and pelvic joint movements during gait and look for differentiating areas with an increased level of asymmetry of the gait cycle in individuals who underwent an AKA and are equipped with MicPK or MechPK.

**Methods:**

Twenty-eight individuals participated in the study; 14 were assigned to a study group of individuals who underwent a unilateral AKA, and the other 14 were healthy participants as a control group. The movement task was recorded using the optoelectronic SMART-E system following the standard Davis protocol (the Newington model). A new method of quantifying gait symmetry using the symmetry function (SF) is proposed. SF is an integral measure of absolute differences in time-standardized signals between sides throughout the whole cycle of motion variability.

**Results:**

In the frontal plane, there were significant differences between groups in the asymmetry of the range of movement in the hip joint of the intact limb. In the middle of the support phase, the intact limb was more adducted in individuals with MicPK and less abducted in people with MechPK (differences in mean 180%, p < 0.000; max 63%, p < 0.000; min 65%, p < 0.000). In the sagittal plane, the range of asymmetry of the flexion and thigh extension of the intact limb was similar to and only slightly different from the physiological gait. In the transverse plane, higher asymmetry values were noted for individuals with MicPK. In the final stage of the swing phase, the thigh was more rotated both externally and internally. The size of the asymmetry, when compared to gait of healthy individuals, reached 50% (differences in mean 115%, p < 0.232; max 62% p < 0.26; min 50, p < 0.154).

**Conclusions:**

In the study group, the assessed ranges of pelvic and thigh movement in the hip joint differed only in the frontal plane. Individuals who underwent a unilateral above-knee amputation may gain less from using MicPK than anticipated.

## Introduction

The percentage of above-knee amputations (AKAs) performed for reasons other than vascular has remained constant over the years and reached 0.92 amputations per 1000 people [1, 2]. A significant number of affected individuals do not have any additional major comorbidities affecting the musculoskeletal system [3], which allows them to achieve good daily performance results when using a prosthesis [4]. However, AKA contributes to gait asymmetry [5]. Increased levels of gait asymmetry may not merely adversely affect the joints; those individuals who undergo a unilateral amputation are also predisposed to flexion contracture of the thigh residual limb (especially in a short residual limb), osteoarthritis of the contralateral limb [6], and more frequent development of lower back pain (LBP) [7, 8]. Therefore, a significant number of potential health-related outcomes resulting from an asymmetrical gait are worthy of in-depth examination.

Increased levels of gait asymmetry usually occur in clinical populations such as individuals with lower limb amputations. These asymmetries are often considered to be a consequence of a pathology in which a patient compensates for movements to avoid pain or because of the desire to align the asymmetrical gait pattern [9]. According to research, the degree of gait symmetry of individuals who underwent unilateral AKA is influenced by the cause of amputation [10, 11], level of physical activity [12, 13] or type of prosthesis [14–17]. The key elements of the lower limb prosthesis are a well-fitted prosthetic socket and prosthetic knee joint module. With AKA, correctly choosing the prosthetic knee joint is important for safety and quality of movement [18]. The prosthetic limb may perform significantly more eccentric hip flexor work than the normal limb because the lack of prosthetic knee flexion is associated with a lack of hip flexion in an early stance, especially when compared to the intact limb [19].

The gait analysis of individuals with a unilateral AKA indicated changes in the range of movement mainly in pelvic tilt, pelvic obliquity, and hip abduction [20]. Knee prosthetics are currently described as mechanical modules (MechPK) or microprocessor-controlled modules (MicPK). The main difference in operation between MechPK and MicPK lies in the control of the swing and stance phases. MechPK only provides swing or stance phase control with manual locking, constant friction, weight-activated friction, and mechanisms that could increase stability in the stance phase. MicPK, on the other hand, is equipped with load and position sensors, which enables a more complete imitation of knee movements. In available studies, individuals with MicPK were characterized by a smaller asymmetry of thigh movements in the hip and knee joints [21, 22] and better balance [23]. However, there are reports suggesting that MechPK predominates over MicPK among patients [24].

Although some scalar indicators are a measure of space–time symmetry, they still do not provide a comprehensive assessment of overall gait symmetry. It has been observed that asymmetry of the step length (using the symmetry index, SI the relative difference between sides) for individuals who underwent an amputation differed between participants, and the results suggested symmetry for some even though their overall gait was asymmetrical [25, 26]. Therefore, a single measurement cannot provide extensive information on gait symmetry because asymmetries in the course of angle variations (angle-time characteristics), from which those metrics are derived, are not well described by numerical values. To accurately assess gait symmetry, the behavior of a whole limb (or a limb in a prosthesis) throughout the entire gait cycle should be considered. This suggests the need for more complete methods of measuring symmetry.

Conducting research that aims at detecting areas of increased differences between lower limbs in the gait of individuals who underwent AKA should optimize gait re-education. The limb amputation protocols for rehabilitation stages very often assume that supplies are selected based on progress in physiotherapy. However, this decision is usually based on the experience of the treatment team rather than on empirical data. We adopted the hypothesis that individuals equipped with MicPK knee will have better gait symmetry results than people with MechPK. The available literature offers only a limited number of studies presenting the results of such analysis [27].

Therefore, this study aimed at evaluating kinematic parameters of pelvic and hip joints in gait and looking for differentiating areas with an increased level of gait cycle asymmetry, compared to a normal gait, in individuals who underwent AKA and were equipped with MicPK or MechPK. The following research questions to be addressed: (1) Is the range of pelvic and thigh movements in the hip joint in the gait cycle of people equipped with MicPK different from that in individuals with MechPK? (2) Are there any characteristic adaptive variations between using prostheses with MicPK and MechPK expressed in the magnitude of gait cycle asymmetry? (3) Are there any differences in the gait cycle compared to the normal values (gait of healthy individuals) that support the use of MicPK in individuals who underwent unilateral AKA?

## Methods

### Study design, settings, and participants

Twenty-eight individuals participated in this cross-sectional study; 14 were assigned to an experimental group (individuals who underwent unilateral AKA), and the other 14 were healthy participants as a control group (Table 1). The control group showed no differences in terms of somatic structure and also representative of the population of middle-aged women and men.

**Table 1:** Demographic characteristics of study participants (mean ± SD)

Characteristics	TFA (n = 14)	Healthy (n = 14)
Age [years]	41.5 ± 15	32.3 ± 4
Male/female	12/2	8/7
Body mass [kg]	79.6 ± 20	80.1 ± 9
Height [cm]	176.2 ± 0,1	177.4 ± 10
BMI [kg/m^2^]	25.70 ± 6.3	25.57 ± 2.1

*TFA* trans femoral amputation, *SD* standard deviation, *n* number of participants, *BMI* body mass index

This study was approved by the Bioethics Committee at the Medical University in Wroclaw (approval no. KB–232/2016). Informed verbal and written consent to take part in this study was obtained from each participant. The study was carried out in accordance with the tenets of the Declaration of Helsinki and Good Clinical Practice guidelines. This study adhered to the STROBE (Strengthening the Reporting of Observational Studies in Epidemiology) guidelines for observational studies.

### Qualification procedure

Inclusion criteria were as following: (1) being at least 18 years old on the day of the examination, (2) having a reason other than vascular for the amputation, (3) using current prosthetic equipment on a daily basis for at least 6 months prior, and (4) the ability to walk at least 30 m without any help (Medicare functional classification level 3 or 4).

Exclusion criteria were as following: (1) a history of musculoskeletal disorders that cause pain, (2) fatigue, (3) a reduced range of lower limb movement or loss of coordination, (4) a dysfunction of the neuromuscular, cardiovascular or respiratory systems, and (5) cognitive impairments. Seven individuals equipped with single-axis hydraulic C-Leg were assigned to the MicPK group. The remainder of the participants were equipped with hydraulic polycentric knee unit 3R80 or 3R95 (without the microprocessor control) and assigned to the MechPK group. Other clinical and demographic characteristics are presented in Tables 1 and 2.

**Table 2:** Clinical characteristics in above-knee amputees

No	Side	Cause	Body height [m]	Residual limb length (cm)	Socket type	Prosthetic knee	Prosthetic foot
P1	R	Trauma	1.71	29.5	ICS	C-Leg®	1C40 C-Walk®
P2	L	Trauma	2.00	35	ICS	C-Leg®	1C40 C-Walk®
P3	L	Trauma	1.78	29	MAS	3R80®	1C30 Trias®
P4	L	CM	1.83	34	ICS	3R95®	1C30 Trias®
P5	R	Trauma	1.82	21	ICS	C-Leg®	1C40 C-Walk®
P6	R	Cancer	1.71	22	ICS	C-Leg®	1C40 C-Walk®
P7	L	Trauma	1.75	24	ICS	3R80®	1C30 Trias®
P8	L	Infection	1.68	27	ICS	3R80®	1C30 Trias®
P9	L	Cancer	1.64	22.5	ICS	C-Leg®	1C60 Triton
P10	L	CM	1.70	24	ICS	3R95®	1C30 Trias®
P11	R	Cancer	1.70	25.5	MAS	3R80®	1E56 Axtion®
P12	L	Trauma	1.83	32	ICS	C-Leg®	1C60 Triton®
P13	R	Trauma	1.63	28.5	ICS	C-Leg®	1C60 Triton®
P14	R	Cancer	1.81	25	ICS	3R95®	1C40 C-Walk®

*CM* congenital malformation, *MAS* Marlo Anatomical Socket, *ICS* ischial containment socket

### Measurement conditions

The study was conducted in a certified Biomechanical Analyses Laboratory of the Department of Biomechanics at the Academy of Physical Education in Wroclaw (Poland) between June and September 2019. The movement task was recorded using the optoelectronic SMART-E system (BTS Bioengineering, Milan, Italy) following the standard Davis protocol (the Newington model). The system consisted of six infrared (IR) cameras detecting reflecting markers with a sampling frequency of 120 Hz. Twenty-two markers were placed above the following anatomical points on participants’ skin: C7, the acromion processes, sacrum, anterior superior iliac spines (ASIS), greater trochanters, thighs (midpoint between the greater trochanter and lateral head of the femur), knees (lateral head of the femur and fibula), shank (midpoint between the lateral head of the fibula and the lateral malleolus), lateral malleoli, second metatarsal heads, and heels.

The measuring station was equipped with Smart Capture software for data recording, Smart Tracker for tracking the filming markers, and Smart Analyzer for data analysis and processing. The computer’s central processing unit contained a video controller (System VIX) and three network hubs (one 32-channel analog hub and two digital hubs, each with four communication ports). The IR cameras were rigidly fixed on the lab walls via frames [28].

### Measurement procedure

Participants were asked to walk a distance of approximately 6 m both ways three times at their preferred walking speed, which allowed for an isolation of eight cycles for both lower limbs (measured together).

The individual biomechanical assessment included measuring the angle-time relationship characterizing the range of motion (ROM) in the lower limbs’ joints, especially (1) the pelvic obliquity angle (up/down)—rotation of the mediolateral axis out of the horizontal plane, where a positive value (up) corresponds to anterior superior iliac spine (ASIS) and posterior superior iliac spine (PSIS) markers that are higher than the corresponding markers on the contralateral side; (2) the pelvic tilt angle (up/down)—anterior/posterior rotation around the mediolateral axis, where a positive value (up) corresponds to the normal situation in which the PSIS is higher than the ASIS; (3) pelvic rotation angle (internal/external)—rotation of the mediolateral axis around the vertical axis; (4) the hip ad/abduction angle—rotation of the proximal–distal axis out of the sagittal anatomical plane; (5) the hip flexion–extension angle—rotation of the proximal–distal axis around the mediolateral axis, where a positive (flexion) angle value corresponds to the knee being in front of the body; (6) the hip rotation angle (internal/external)—rotation around the proximal–distal axis; and (7) the knee flexion–extension angle—rotation of the proximal–distal axis around the mediolateral axis, where a positive angle corresponds to a flexed knee [29, 30].

The following parameters were extracted: mean value, peak positive and negative values (Peak + and Peak −), and ROM calculated from the angle-time characteristics to statistically compare the differences. The gait cycle began when a limb first contacted the ground (at 0% GC). Based on the mathematical formula of *SI* [31], we propose a method of quantifying the gait symmetry with a help of the symmetry function (SF), which is an integral measure of absolute differences in time-standardized signals between the sides throughout the whole cycle of motion variation [32].

$${\text{SF}}(t) = 2 \cdot \frac{{x_{{{\text{un}}}} \left( t \right) - x_{{{\text{in}}}} \left( t \right)}}{{{\text{Range}}\left( {x_{{{\text{un}}}} (t)} \right) + {\text{Range}}\left( {x_{{{\text{in}}}} (t)} \right)}}$$,

where *x*_un_ and *x*_in_ are any variables in function of time (time series) for the uninvolved and involved sides and Range is a range of variation for those characteristics.

Because a function constructed this way can combine variables of different physical dimensions, the difference signals have been normalized to the appropriate average range of their variability. SF functions have been grouped according to the relevant plane of movement (or movement degree of freedom), as in the case of angular waveforms, to track the process of physiotherapy in gait re-education.

### Statistical analysis

Descriptive statistics such as Mean, Min, Max, and Range were carried out for (a) angle variation, (b) symmetry function (SF) between the left and right side, (c) SF between the TFA.UN limb and a intact limb of the control group (Norm), as well as, (d) SF between TFA.IN limb and a intact limb of the control group (Norm) based on the type of the knee prosthesis (C-Leg / Mech). The non-parametric methods were used for comparison the knee prosthesis (C-Leg / Mech) and parametric test were used for group comparison (i.e., AKA vs. healthy). Collected parameters were tested for statistically significant differences using a normality test (Kolmogorov–Smirnov test) as well as a test for the equality of group variances (Brown-Forsythe test). The significance of differences between groups was measured using one-way ANOVA with Bonferroni post-hoc test (for variables with distribution close to the normal distribution) and non-parametric Mann–Whitney U test with continuity correction (for variables that deviate from the normal distribution) with the assumed significance level alpha = 0.05. All statistical procedures were carried out using Statistica 13.1 software (TIBCO Software Inc., Palo Alto, CA, USA).

## Results

The main speed (and ± 1 standard deviation) for the MicPK group was 0.97 ± 0.10 (m/s) and 0.91 ± 0.08 (m/s) for the MechPK group. There was no statistically significant difference between the speeds of test groups. The course of change in the angle of the pelvic movement in the gait cycle on each side was characterized by similar values in both the MechPK and MicPK groups (Table 3).

**Table 3:** The mean, minimum, maximum, and range of angles characterizing pelvic movements in frontal (POBLI), sagittal (PTILT), and transverse (PROT) plane as well as hip joint movements in frontal (HPAA), sagittal (HPFE), and transverse (HPROT) plane and knee joint movements sagittal plane (KFE) of an amputated limb (in) and an intact limb (un)

		Mean		Min		Max		Range	
		MicPK	MechPK	MicPK	MechPK	MicPK	MechPK	MicPK	MechPK
POBLI	in	0.4	1.3	− 5.5	− 4.0	6.0	7.4	11.5	11.4
	un	− 0.4	− 1.4	− 6.1	− 7.4	5.5	4.0	11.6	11.3
PTILT	in	16.2	16.5	10.9	13.0	21.1	19.6	10.2	6.6
	un	16.2	16.4	10.8	13.0	21.2	19.5	10.4	6.5
PROT	in	2.7	2.1	− 4.1	− 3.7	9.6	8.4	13.7	12.1
	un	− 2.8	− 2.0	− 9.9	− 8.2	4.1	3.6	14.0	11.8
HPAA	in	− 4.8	− 8.8	− 10.6	− 14.0	1.8	− 3.6	12.4	10.5
	un	− 5*	0.7 ^Δ^	− 10*	− 2.8 ^Δ^	− 0.6	4.6	9.4	7.4
HPFE	in	20.9	20.7	− 4.7	− 2.4	44.9	41.3	49.7	43.7
	un	20.8	19.6	− 1.4	− 2.1	39.0	38.8	40.4	41.0
HPIE	in	1.2	3.9	− 5.8	− 4.0	7.8	12.2	13.5	16.2
	un	− 5.7	− 2.4	− 12.3	− 11.4	2.1	6.1	14.5	17.5
KFE	in	14.4	14.3	− 2.3	− 0.2	57.3	51.5	59.7	51.8
	un	15.7	12.4	− 2.6	− 3.1	57.1	49.8	59.6	52.9

^*^Statistically significant differences; p < 0.05 MicPK

^Δ^Statistically significant differences; p < 0.05 MechPK

**Fig. 1:**
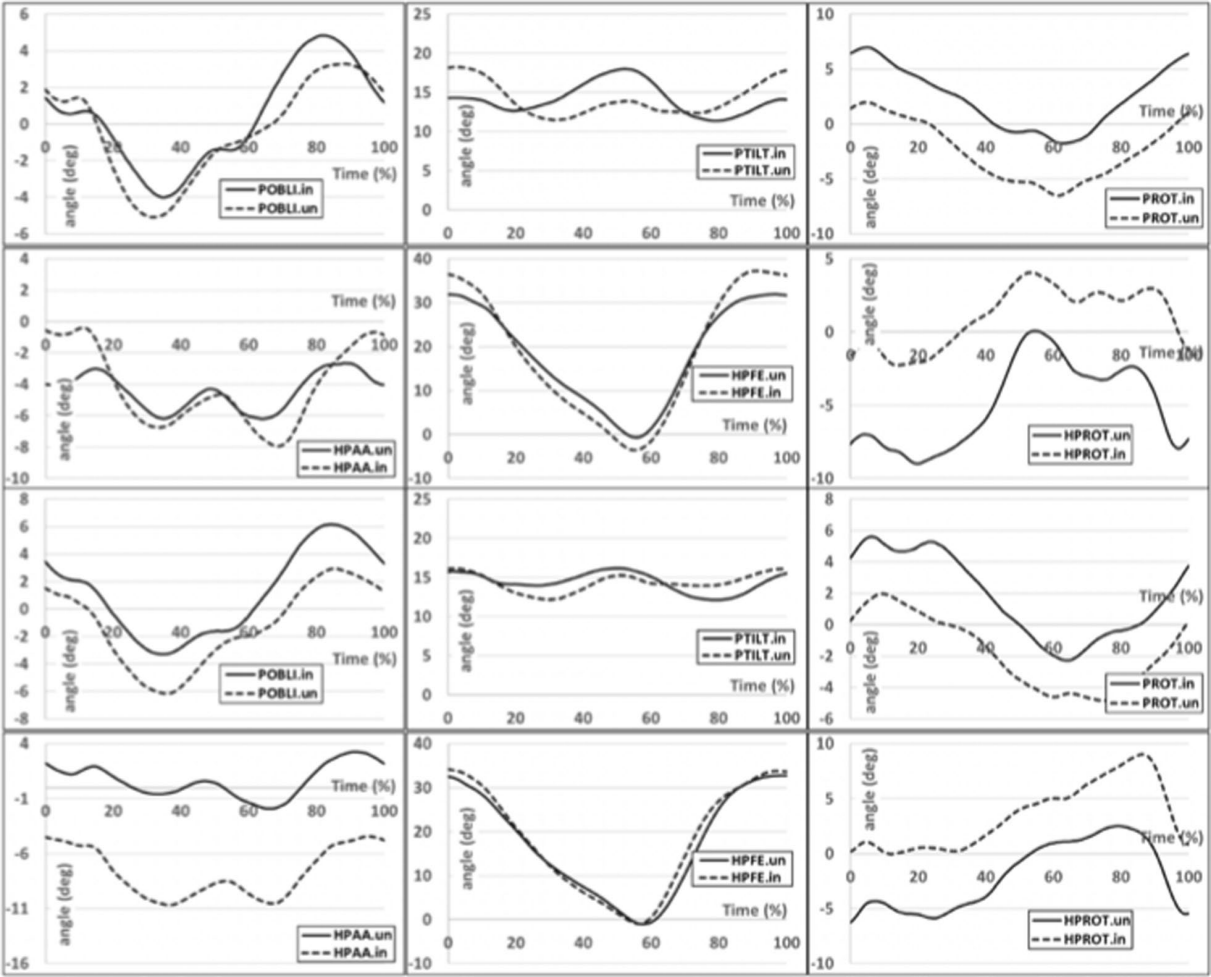
Graphs presenting the angle variation for the function duration of the gait cycle for a prosthetic limb (solid line) and an intact limb (dashed line) for the MicPK group (on the top) and MechPK group (on the bottom) characterizing pelvic movements in frontal (POBLI), sagittal (PTILT), and transverse (PROT) plane as well as hip joint movements in frontal (HPAA), sagittal (HPFE), and transverse (HPROT) plane

The characteristics of thigh movements in the hip joint of the intact limb for the MechPK group significantly differed for the frontal plane when compared with values recorded for the MicPK group (differences in mean 115%, p < 0.00; max 113%, p < 0.056; min 72%, p < 0.00). The thigh of the amputated limb of individuals from the MicPK group was adducted throughout the whole gait cycle. The ranges of motion for both groups and limbs in the sagittal plane were similar. In the transverse plane, the thigh of the amputated limb of those in the MicPK group was rotated externally at the end of the stance phase. For the MechPK group, on the side of the amputated limb, the highest values of the external rotation occurred at the end of the swing phase (differences in mean 70%, p < 0.554; max 65%, p < 0.313; min 8%, p < 1.232) (Fig. 1).

Applying the SF equation for pelvic movements on the side of the amputated limb in the frontal plane determined that the change of the asymmetry values did not significantly differ for either group (Table 4).

**Table 4:** The mean, minimum, maximum, and range values of the symmetry function characterizing pelvic movements in frontal (POBLI), sagittal (PTILT), and transverse (PROT) plane as well as hip joint movements in frontal (HPAA), sagittal (HPFE), and transverse (HPROT) plane and knee joint movements sagittal plane (KFE)

	Mean		Min		Max		Range	
	MicPK	MechPK	MicPK	MechPK	MicPK	MechPK	MicPK	MechPK
POBLI	3.1	17.6	− 30.9	− 8.5	32.7	46.5	63.6	54.9
PTILT	− 0.1	0.7	− 69.5	− 67.9	73.0	70.7	142.4	138.7
PROT	51.3	50.3	12.6	17.4	91.3	86.2	78.7	68.7
HPAA	− 6.5	103.9	− 77.42*	56.5 ^Δ^	43.935*	149.8 ^Δ^	121.4	93.7
HPFE	0.3	− 1.7	− 31.6	− 31.6	28.3	17.4	59.8	48.9
HPIE	− 56.1	− 30.1	− 111.8	− 83.4	6.0	34.2	117.8	117.6
KFE	2.4	− 0.4	− 46.3	− 35.7	46.6	44.9	92.9	80.6

^*^Statistically significant differences; p < 0.05 MicPK

^Δ^Statistically significant differences; p < 0.05 MechPK

**Fig. 2:**
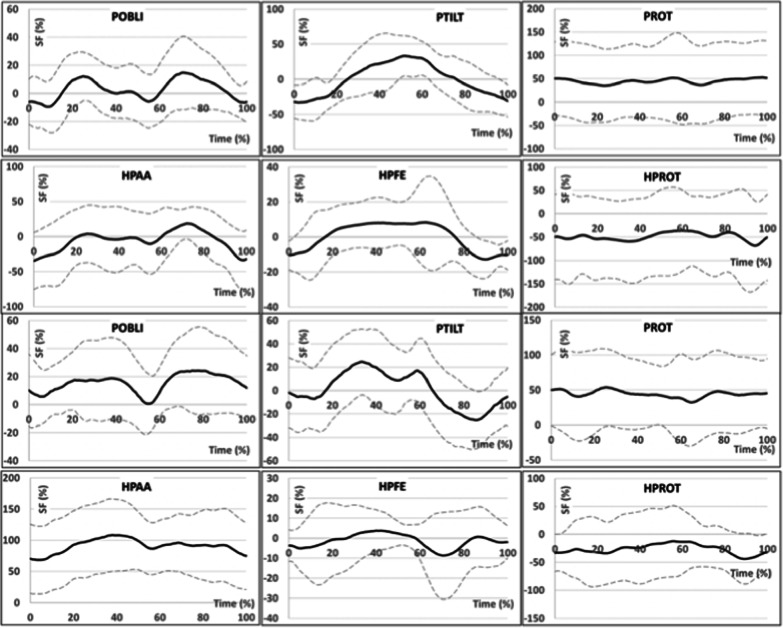
Graphs presenting variability of the axial symmetry function in relation to the duration of the gait cycle for the amputated limb (solid line) ± average standard deviation (dashed line) for MicPK group (on the top) and MechPK group (on the bottom) characterizing pelvic movements in frontal (POBLI), sagittal (PTILT), and transverse (PROT) plane as well as hip joint movements in frontal (HPAA), sagittal (HPFE), and transverse (HPROT) plane

The highest asymmetry values of pelvic movements in the sagittal plane occurred in the stance phase for both groups. The pelvis was tilted forward for longer in the MechPK group. The asymmetry of pelvic movements in the transverse plane occurred throughout the whole gait cycle for both groups. The pelvis was turned toward the contralateral limb (differences in mean 83%, p < 0.376; max 126%, p < 0.387; min 73%, p < 0.354) (Fig. 2).

**Fig. 3:**
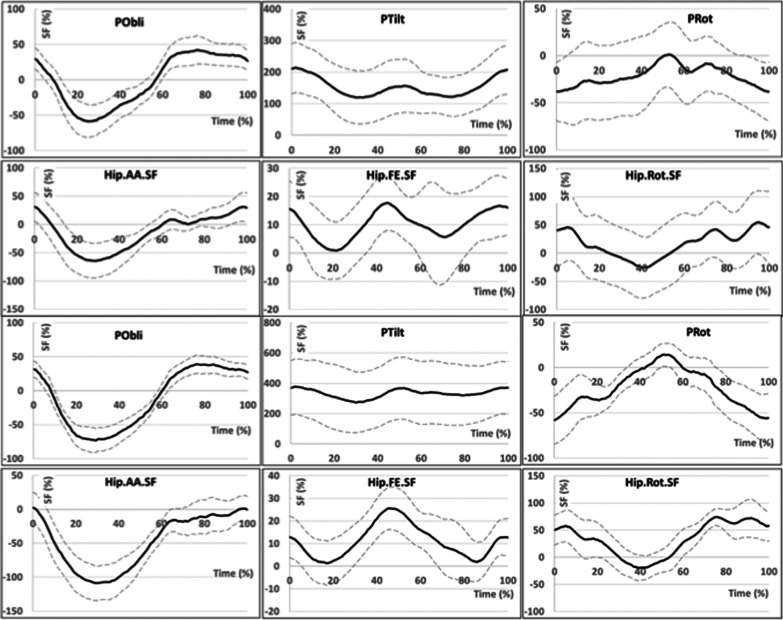
Graphs presenting variability of the symmetry function (relative to the norm) in relation to the duration of the gait cycle for the amputated limb (solid line) ± average standard deviation (dashed line) for the MicPK group (on the top) and MechPK (on the bottom) characterizing pelvic movements in frontal (POBLI), sagittal (PTILT), and transverse (PROT) plane as well as hip joint movements in frontal (HPAA), sagittal (HPFE), and transverse (HPROT) plane

Statistically significant differences in asymmetry of thigh movements in the hip joint of the amputated limb occurred in the frontal plane. In the MicPK group, the thigh was characterized by lower adduction asymmetry values and higher asymmetry values in the transverse plane and was rotated internally throughout the whole gait cycle. In the MechPK group, the thigh was more abducted throughout the whole gait cycle and less internally rotated. The ranges of asymmetry of thigh movements in the sagittal plane were similar, and the difference between groups in the size of the asymmetry occurred mainly in the swing phase (differences in mean 106%, p < 0.03; max 71%, p < 0.00; min 172%, p < 0.00) (Fig. 3).

On the side of the amputated limb, the course of the range of pelvic movement asymmetry variability in the frontal plane of the gait cycle presented similar values for both groups (Table 5). The range of asymmetry compared to the movements of healthy individuals was the highest in the middle stance and swing phases when the pelvis on the side of the amputated limb was lowered in both groups (differences in mean 89%, p < 0.329). In the sagittal plane, for both MechPK and MicPK, the pelvis on the side of the amputated limb was tilted forward throughout the entire gait cycle. Higher asymmetry values were reported in the MechPK group, where the level of asymmetry ranged from 300 to 400%, whereas in the MicPK group, it ranged from 110 to 200% (differences in mean 47%, p < 0.353; max 38%, p < 0.402; min 64%, p < 0.352). In the transverse plane, the pelvis was rotated toward the contralateral direction at the beginning of the stance phase for both groups. The size of SF in the gait cycle for the amputated limb did not significantly differ between the groups.

**Table 5:** The mean, minimum, maximum, and range values of the variability of the symmetry function (relative to the norm) characterizing pelvic movements in frontal (POBLI), sagittal (PTILT), and transverse (PROT) plane as well as hip joint movements in frontal (HPAA), sagittal (HPFE), and transverse (HPROT) plane and knee joint movements sagittal plane (KEF) of an amputated limb (in) and an intact limb (un)

		Mean		Min		Max		Range	
		MicPK	MechPK	MicPK	MechPK	MicPK	MechPK	MicPK	MechPK
POBLI	in	− 1.2	− 11.6	− 70.3	− 78.5	58.5	49.9	128.8	128.4
	un	1.7	10.8	− 57.9	− 53.4	73.8	77.9	131.6	131.3
PTILT	in	175.1	333.3	89.6	248.5	256.0	409.7	166.4	161.2
	un	181.7	283.8	94.6	200.6	264.2	362.9	169.6	162.4
PROT	in	− 24.4	− 22.7	− 64.7	− 69.6	14.7	29.9	79.4	99.5
	un	24.4	23.1	− 12.1	− 23.7	67.9	70.9	79.9	94.7
HPAA	in	− 16.1	− 48.3	− 78.0	− 112.9	45.4	14.1	123.4	127.1
	un	− 26.6*	− 33.3 ^Δ^	− 87.85*	− 31 ^Δ^	35.1*	96.1 ^Δ^	123	127.2
HPFE	in	11	11.6	− 8.4	− 11.2	31.9	32.6	40.3	43.7
	un	13.6	9.5	− 8.0	− 15.6	37.3	33.1	45.3	48.7
HPIE	in	18.8	33.1	− 33.3	− 27.5	85.7	98	119.1	125.5
	un	− 36.2	5.5	− 97.5	− 48.7	25.5	67.1	123	115.9
KFE	in	− 15.6	− 19.8	− 48	− 57.9	13.7	6.2	61.7	64.1
	un	− 13.6	− 21.5	− 42.1	− 48.1	8.3	− 0.4	50.4	47.7

*Statistically significant differences; p < 0.05 MicPK

^Δ^Statistically significant differences; p < 0.05 MechPK

**Fig. 4:**
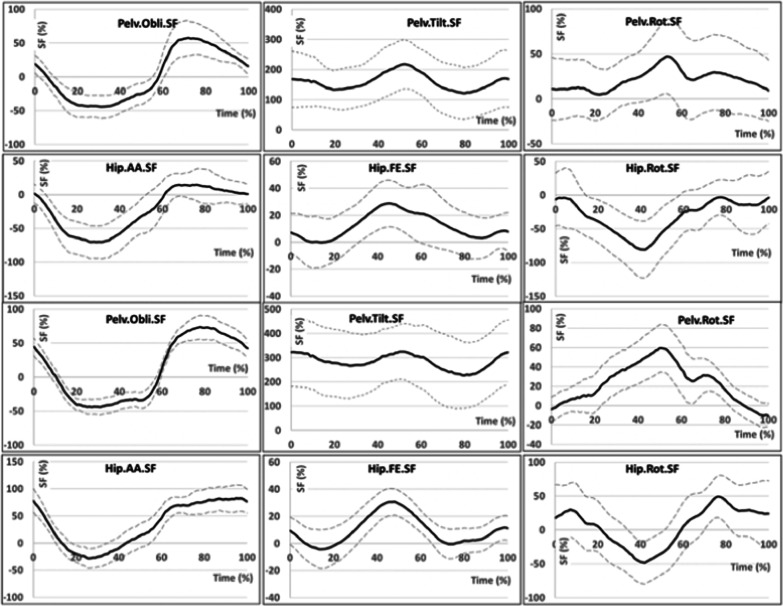
Graphs presenting variability of the symmetry function (relative to the norm) in relation to the duration of the gait cycle for the intact limb (solid line) ± average standard deviation (dashed line) for the MicPK group (on the top) and MechPK (on the bottom) characterizing pelvic movements in frontal (POBLI), sagittal (PTILT), and transverse (PROT) plane as well as hip joint movements in frontal (HPAA), sagittal (HPFE), and transverse (HPROT) plane

The level of thigh movement asymmetry in the hip joint of an amputated limb in the frontal plane differed between the measured groups in the stance phase. In the MicPK group, the thigh was positioned in greater abduction at the beginning of the stance phase than it was in the MechPK group. In the middle of the stance phase, the level of asymmetry was greater for individuals in the MechPK group. Moreover, in this group, the thigh in the hip joint was more adducted. The level of asymmetry compared to healthy individuals reached 100% (differences in mean 67%, p < 0.124; max 69%, p < 0.125; min 65%, p < 0.123). In the sagittal plane, the range of flexion and extension movement asymmetry of the amputated limb was similar and only slightly different from the physiological gait. In the transverse plane, the thigh of the amputated limb was rotated externally at the beginning of the stance phase. The level of asymmetry compared to healthy individuals reached 50% (Fig. 4).

The variability of angular values after applying the SF equations for pelvic movements of the intact limb in the frontal plane in the stance phase was similar for both groups. The largest differences compared to pelvic movements in the frontal plane for healthy individuals occurred in the swing phase, when the pelvis was positioned nearly 50% higher. In the sagittal plane, similar to pelvic movement asymmetry on the side of the amputated limb, the pelvis was tilted forward on the side of the intact limb throughout the whole gait cycle (differences in mean 36%, p < 0.383; max 27%, p < 0.404; min 53%, p < 0.372). The level of asymmetry was similar to that of the amputated limb, higher for the MechPK group (200–300%) than for the MicPK group (100–200%). Pelvic movements in the transverse plane were characterized by the highest asymmetry values in the final stage of the stance phase. The pelvis was rotated toward the contralateral limb. The size of the SF in the gait cycle for the amputated limb did not significantly differ between groups.

Statistically significant between-group differences in the asymmetry of thigh movement range in the hip joint of the intact limb were found in the frontal plane. The intact limb in the middle of the stance phase was more adducted in the MicPK group and less adducted in the MechPK group (differences in mean 180%, p < 0.000; max 63%, p < 0,000; min 65%, p < 0.000). In the sagittal plane, the range of asymmetry of the flexion and extension of the thigh of the intact limb was similar and only slightly different from the physiological gait. In the transverse plane, higher asymmetry values were observed for individuals from the MechPK group. The thigh was more externally rotated at the end of the swing stage and more internally rotated in the swing phase. The level of asymmetry compared to the gait of healthy individuals reached 50% (differences in mean 115%, p < 0.232; max 62%, p < 0.26; min 50, p < 0.154).

## Discussion

Starting the rehabilitation of individuals who underwent lower limb amputations is difficult. Amputation for reasons other than vascular is often done to save the patient’s life. Individuals then entrust considerable hope in effective and optimal prosthetics, in which they see a possibility of returning to their previous lifestyle. Choosing the right type of prosthesis is an important and key stage, therefore this research might help to making the appropriate choice of prosthetic.

Humans’ symmetrical gait pattern is characterized by an almost identical course on both sides of the body. This symmetry usually deteriorates into a pathological gait due to pain or disturbances in the normal function of the locomotive system resulting from AKA. Over time, the persistence of the asymmetrical gait pattern can lead to other musculoskeletal dysfunctions. Based on this assumption, we conducted research aimed at confirming the existence of differences in gait asymmetry in the population after AKA, in relation to the type of prosthetic supplies. We also focused on the assessment of effectiveness and the feasibility of using the SF tool to diagnose gait symmetry.

Asymmetry might be investigated from the point of view of time-spatial parameters (e.g., uneven walking lengths) and angular parameters (e.g., a difference in the ROM between the amputated and sound limbs). The differences in ROM varied between experimental subgroups in the frontal plane in the hip joint of the intact limb. Seroussi et al. [19] presented similar results for the ankle, knee, and hip: 5.6, 6.3, and 5.5 degrees, respectively, for the prosthetic limb and 6.6, 4.7, and 7.3 degrees, respectively, for the intact limb. The range of hip movements in the sagittal plane in individuals from the experimental group was similar to the normal gait value. Statistical analysis did not confirm other differences in the gait cycle between the MechPK and MicPK groups. Kaufman et al. [16] also reported that the range of movements in the sagittal plane in both amputated and intact limbs was similar in compared groups of individuals after AKA equipped with MechPK and MicPK. These results allow us to conclude that both prosthetic modules correctly imitate physiological knee movements, but the time of the intensity of asymmetry in the gait cycle differs.

The proposed method identifies where in the gait cycle the asymmetry is less or more pronounced. The suggested measure of symmetry measures not only the overall symmetry in each component of movement but also explains whether the asymmetry is caused by a shift in time or difference in values. The thigh of individuals in the MechPK group was abducted throughout the whole gait cycle and externally rotated. The thigh of those in the MicPK group was also abducted, but the degree of asymmetry was lower. A higher degree of asymmetry occurred in external rotation movements. These results may indicate a different degree of adaptation for people who underwent AKA, resulting from a difference in knee prosthetic modules’ performance. Kaufman et al. [21] state that the gait of individuals equipped with MicPK is similar to the correct one. However, our research shows that the gait of people with MicPK might be more similar to the correct gait in the frontal plane, whereas the degree of asymmetry in the transverse plane is greater than that of people with MechPK. This also influences an uneven load distribution, which results from an asymmetrical gait pattern regardless of module type [33]. Luetmer et al. [8] and Devan et al. [34] state that the type of prosthetic does not reduce the frequency of LBP, confirming this idea.

Unlike the known scalar-based measures of symmetry, a function-based measure quantifies symmetry over the entire gait cycle and, in certain cases, at numerous levels of analysis. Time waveforms can be based on kinematic or kinetic variables, ground reaction forces, or muscle activity patterns that are available as a result of motion analysis [32]. The highest values of pelvic movement asymmetry compared to healthy individuals occurred in the sagittal plane [35]. Research shows that this result may be influenced by the type of prosthetic socket [36]. Participants in the experimental group used an anatomical prosthetic socket, in which the thigh residual limb is supported on the medial wall or on all walls; one example is the Marlo Anatomical Socket (MAS) type. According to Rabuffetti et al. [37], relative to healthy individuals, those who underwent AKA were characterized by a reduction of extension in the hip joint on the side of the amputated limb of 7.5 ± 4.4 degrees, an increase in pelvic inclination of 8.2 ± 3.6 degrees, and increase in hip joint flexion on the side of the intact limb of 13.8 ± 7.4 degrees. Participants in the experimental group had a different pattern of asymmetry regardless of the prosthetic socket. The efficiency of the use of a lower limb prosthesis largely depends on the length the thigh stump, muscle strength and flexibility of the surrounding tissues. Previous studies have confirmed the impact of the generated moment of force on the gait pattern of individuals after AKA [32, 38]. Therefore, the differences in the use of lower limb prostheses can be observed in the efficiency of the thigh residual limb.

The number of publications on gait asymmetry in clinical trials is increasing due to the potential implications. Individuals after AKA in a gait re-education program can use a simple measure of symmetry that can be easily and quickly interpreted. This suggestion is confirmed by research showing that learning and maintaining complex motor skills (such as a more symmetrical gait pattern) is reinforced by external feedback associated with the overall assessment of locomotion performance rather than specific movements. In this study, different strategies were observed for a lower limb prosthesis depending on the type of knee module.

### Study limitations

The present study has some potential limitations. First, the research was carried out in the clinical conditions of only one center. Second, the size of the AKA group was not large and was insufficiently representative to generalize changes in differences in the range of movements in individuals equipped with MechPK and MicPK to the whole population of individuals after unilateral AKA. Finally, the research does not concentrate on the relationship between gait analysis outcomes and the physical characteristics of the participants.

### Practical implications

When choosing the final prostheses for individuals with unilateral AKA, it is important to first assess the condition and functional efficiency of the thigh residual limb. Furthermore, less technically advanced prostheses are similar to the more advanced solutions in allowing for restoring a pattern of the gait cycle. The type of prosthetic equipment should be considered, due to differences in gait movement compensation, especially in the frontal plane.

## Conclusions

The results of this study allowed for the following conclusions to be drawn. MicPK reduces the asymmetry values when compared to the movements of healthy individuals, especially the movements in the sagittal plane and pelvic rotations. Participants equipped with MechPK and MicPK develop a different type of gait in the prosthesis, expressed by changing the size of the asymmetry of the pelvic and thigh movements in the hip joint. Furthermore, in the experimental group, the kinematic variables of pelvic and thigh movements in the hip joint differ only in movements in the frontal plane. Individuals who underwent unilateral AKA can gain less from using an MicPK lower limb prosthesis than was anticipated. Our observations show, both in people with MicPK and MechPK protheses, the degree of adduction of the thigh in the hip joint during the support phase is increased. This results in the need for greater involvement of the abduction muscles of the hip joint (mainly the gluteus medius muscle). This study shows that the choice of module in the final prosthesis should be based on the size of changes in the thigh stump and depend on the ability to control the prosthesis in frontal plane individuals with short limb stumps.

## Data Availability

The datasets used and/or analysed during the current study are available from the corresponding author on reasonable request.

## Abbreviations

AKA: Above-knee amputation
ASIS: Anterior superior iliac spines
IR: Infrared
MAS: Marlo anatomical socket
MechPK: Mechanical modules
MicPK: Microprocessor modules
PSIS: Posterior superior iliac spine
ROM: Range of motion
SF: Symmetry function
STROBE: Strengthening the reporting of observational studies in epidemiology guidelines
